# Philadelphia Hospital

**Published:** 1858

**Authors:** 


### Philadelphia Hospital.

Often and repeatedly have we been called upon to grieve and mourn over the delinquency, if not the manifest want of consistency and principle on the part of medical men who occupy or desire to hold prominent positions in the profession. Never, however, have we felt more chagrin, or suffered from the sting of mortification so painfully as when perusing the history of the above benevolent enterprise, and the outrageous appointment to the Superintendency thereof, of a renegade to the profession, Dr. James McClintock; who, for the past four years has been engaged in the mean pursuit and low traffic of patent nostrums. He had been a teacher of Anatomy and Surgery in a young Institution of that city, and had acquired much celebrity as a lecturer. In the face of these facts and in the very face and eyes of a large body of the most learned men of the country, he basely deserted his standard, joined the band of pirates and plunderers upon human health and life, and brought opprobrium and dishonor upon the profession in which he had figured conspicuously. Avarice and greed were doubtless the prompting motives, nor could the plea of ignorance be set up as an apology. It required no small amount of courage on his part, to perpetrate the outrage, which, in its exercise, exhibited a disregard and contempt for public opinion.

Now he has been recently placed in charge of the Blockley Hospital, notwithstanding his heresy and abandonment of principle, when there are one hundred gentlemen in that Athens in medicine, more honorable and more capable than he for the position, and who have been insulted by this selection. But what adds to the outrage, it appears he was recommended to the place by one who is a member of the A. M. A., beside other medical men of that city. What can these say in extenuation of their unparalleled course? What regard have they for a profession, when they, by their influence, have elevated a man to an honored place in the profession, who has been engaged in the manufacture as well as in vending empirical remedies? The plea of *personal friendship* has been presented as an apology. We were about to say a plea of *fiddlesticks!!* Did not these very men, as well as Dr. McClintock, before the Degree of M. D. was conferred, solemnly covenant and agree that they never would make or vend any patent nostrum, or any quack compound, nor in any way countenance the same? If so, where is their *vow* we ask? Broken and shamefully violated.

We care not if Dr. McClintock has in deep penitence confessed his fault. He should have shown his sense of contrition by a long adherence to those principles which he basely abandoned, and to the interests and honor of a profession he has abused and insulted, before he would seek a position of prominence and influence. The place must be *changed* for a better, and we think this has been the *only change*. We hope this bold act of effrontery will be reverberated by the press and the profession as it deserves; and we here take occasion to endorse the course pursued by the gentlemen who resigned their connexion with the Hospital, however desirable for clinical observation and instruction, rather than be degraded by the association and intercourse with a charlatan. We have no compromises with such.

